# Sub-lethal effects of dietary neonicotinoid insecticide exposure on honey bee queen fecundity and colony development

**DOI:** 10.1038/srep32108

**Published:** 2016-08-26

**Authors:** Judy Wu-Smart, Marla Spivak

**Affiliations:** 1 University of Nebraska-Lincoln, Entomology, Lincoln, 68583, USA; 2 University of Minnesota, Entomology, Saint Paul, 55108, USA

## Abstract

Many factors can negatively affect honey bee (*Apis mellifera* L.) health including the pervasive use of systemic neonicotinoid insecticides. Through direct consumption of contaminated nectar and pollen from treated plants, neonicotinoids can affect foraging, learning, and memory in worker bees. Less well studied are the potential effects of neonicotinoids on queen bees, which may be exposed indirectly through trophallaxis, or food-sharing. To assess effects on queen productivity, small colonies of different sizes (1500, 3000, and 7000 bees) were fed imidacloprid (0, 10, 20, 50, and 100 ppb) in syrup for three weeks. We found adverse effects of imidacloprid on queens (egg-laying and locomotor activity), worker bees (foraging and hygienic activities), and colony development (brood production and pollen stores) in all treated colonies. Some effects were less evident as colony size increased, suggesting that larger colony populations may act as a buffer to pesticide exposure. This study is the first to show adverse effects of imidacloprid on queen bee fecundity and behavior and improves our understanding of how neonicotinoids may impair short-term colony functioning. These data indicate that risk-mitigation efforts should focus on reducing neonicotinoid exposure in the early spring when colonies are smallest and queens are most vulnerable to exposure.

Honey bees, *Apis mellifera* L., provide pollination services to over 150 different crops worldwide^1^,^2^. In recent years, beekeepers in the US, Canada, and parts of Europe have experienced unsustainably high colony losses^3^,^4^,^5^,^6^ highlighting a serious threat to global food security, agricultural productivity, and trade^7^. A number of factors contribute to managed bee losses, including: *Varroa* mites, bacterial and viral infections, poor nutrition, migratory stress, queen failure, and pesticides^3^,^8^,^9^. Neonicotinoid insecticides are a cause for concern due to their toxicity and pervasive use in agricultural and urban areas worldwide^10^,^11^,^12^,^13^. Currently, there is heavy scrutiny of and debate over the field relevance of laboratory-based results, accuracy of field studies, determination of environmentally-realistic exposure levels and relevant experimental dosages, and the interpretation of reported adverse effects of neonicotinoids on bees, other wildlife, and whole ecosystems^11^,^13^,^14^,^15^,^16^,^17^,^18^.

Neonicotinoids are systemic broad-spectrum insecticides that target sucking and chewing insect pests. These insecticides may translocate, at varying concentrations, to all parts of treated plants including the nectar and pollen. Bees may become unintentionally exposed through dust from seed coatings created during planting and through foraging on contaminated pollen, nectar, water, and sap exudates of treated plants^19^,^20^,^21^. Neonicotinoids are currently registered in over 120 countries and represent 24% (valued at US $2.6 billion) of the global insecticide market as of 2008. Imidacloprid, the first registered active ingredient within the neonicotinoid class is considered “highly toxic” (LD50_oral_: 13 ng bee^−1^)^8^ to bees. In addition, imidacloprid (valued at US $1.1 billion) represents 41.5% of the total neonicotinoid market and is the largest selling insecticide in the world^22^, rendering the potential for exposure to bees high.

In this experiment, concentrations of imidacloprid fed to honey bee colonies were based on plant residue studies and selected to simulate potential exposure on foraging bees collecting contaminated and uncontaminated nectar over a typical bloom period in nature. The lower doses, 10 and 20 ppb, approximate residues that are characteristically found in the nectar and pollen of agricultural crops, such as apples and cucurbit vegetables, that are treated by soil-drench and foliar spray applications following label rates^23^,^24^. However, 10 and 20 ppb may be underestimates, as some crops such as cucurbits can have higher residue levels (60–80 ppb in pollen) when neonicotinoids are applied via drip irrigation, foliar spray, or through transplant water^24^. The higher doses, 50 and 100 ppb, represent residues found in urban landscape plants such as shadbush and rhododendron shrubs (*Amelanchier* spp., *Rhododendron* spp.) and Cornelian cherry (*Cornus mas*), which are treated by soil-drench or trunk injections and can express residues in the ppm range^25^,^26^,^27^. Therefore, the concentrations of imidacloprid treatments represent environmentally relevant exposure rates for bees foraging in both rural and urban settings.

The body of knowledge on the effects of neonicotinoids is vast and includes an increasing number of studies on sub-lethal effects, particularly on neurophysiological and behavioral impairments^28^ including metabolic changes to brain activity, impaired foraging and learning performance, and motor functions in worker honey bees^28^,^29^,^30^. Notably, little is known about the effects of neonicotinoids on queen bees. One study has reported negative effects of neonicotinoids on honey bee queen development and mating success^31^. Other laboratory and field studies on bumble bees have shown that sub-lethal exposure to neonicotinoids (imidacloprid, clothianidin, and thiamethoxam) can reduce queen production and disrupt colony initiation^16^,^32^,^33^,^34^,^35^,^36^. Field studies examining colony-level effects on honey bees also have reported higher queen failure and supercedure rates when colonies were exposed to neonicotinoids^35^,^37^. In those studies the behaviors of the exposed queen bees were not recorded. The queen bee is the only individual in the colony that lays fertilized eggs that develop into worker bees necessary for colony growth and survival. Therefore, it is important to study the potential effects of neonicotinoids on this key reproductive individual and the subsequent indirect effects on colony development.

## Results

### Measurements during chronic exposure (day 1–22)

**Figure 1:**
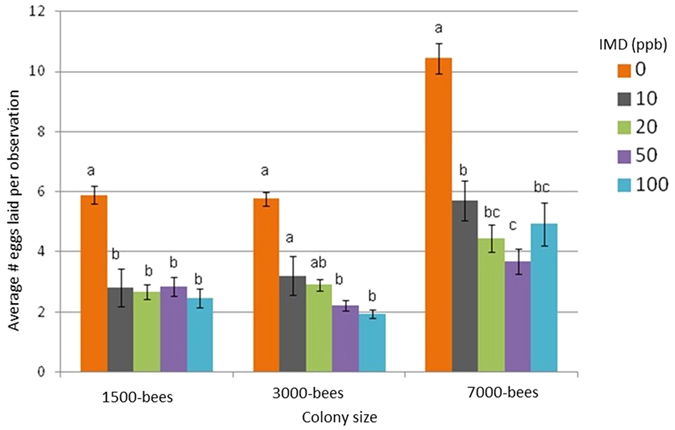
Average (SE) number of eggs laid by queens per 15 minute observation period pooled over three week chronic exposure of imidacloprid (IMD) (0, 10, 20 50, and 100 ppb) in 1500-, 3000-, and 7000-bee colonies ((dose*size*week) interaction: F_16,1053_ = 0.93; p = 0.54; (dose*size) interaction: F_8,1053_ = 6.17; p < 0.0001). Different letters denotes significant statistical differences among treatment levels within each colony size at α < 0.05. Results indicate that queens in untreated colonies laid significantly more eggs than queens in treated colonies at all colony sizes.

Imidacloprid dose (0, 10, 20, 50 and 100 ppb), colony size (1500, 3000 and 7000 bees), and exposure duration (1, 2, and 3 weeks) affected queen behavior. Observed responses occurred within the first week of exposure and effects were sustained throughout the experiment. There was no interaction effect of time with dose and colony size. Time did not consistently or significantly affect queen egg-laying behavior (F_16,1053_ = 0.93; p = 0.54) or activity as measured by the distance travelled and duration of immobility (F_16,2153_ = 1.31; p = 0.18 and F_16,1213_ = 1.65; p = 0.05, respectively). In contrast, there was an interaction effect between dose and colony size for queen egg-laying (F_8,1053_ = 6.17; p < 0.0001), distance travelled (F_8,2153_ = 4.02; p < 0.0001), and duration of immobility (F_8,1213_ = 3.31; p < 0.001) (Fig. 1, Figure S1). Therefore data were pooled over the three-week exposure and separated by treatment dose and colony size in the statistical analysis. Experimental replicates of colony size and treatment dose were established at different times over the season, but there were no effects of start date on any of the measures across the three years (2012–2014).

### Queen egg-laying

Initial worker populations in each colony were equalized by weight, and imidacloprid treatments were randomly assigned. While natural colony variation did occur, pre-treatment measurements showed no significant differences in queen egg-laying rates among colonies of similar sizes (Table S1).

Across colony sizes, there were significant differences in egg-laying rates; as expected queens in 7000-bee colonies laid more eggs compared to queens in colonies with fewer bees (F_2,1053_ = 85.37; p < 0.0001), but there were no differences in egg-laying rate of queens within colonies of 1500 and 3000 bees (F_2,1262_ = 80.56; p = 0.6). Queens in untreated 1500-, 3000-, and 7000-bee control colonies laid on average (SE) 6.5 ± 0.8, 6.0 ± 0.5 and 10.3 ± 0.6 eggs per 15-min observation, respectively, which were significantly (35–65%) more eggs compared to eggs laid in most treated colonies of the same colony sizes (F_4,1053_ = 106.91; p < 0.0001) (Fig. 1). The exceptions were in 3000-bee colonies treated at 10 ppb (2.1 ± 1.3) and 20 ppb (3.2 ± 0.4), where egg-laying rates were not significantly different from rates in control colonies of the same size, due to high variation in queen responses particularly during the first two weeks of exposure (Table S1).

### Queen activity and immobility

Queens in untreated colonies traversed greater distances per observation compared to queens in treated colonies of the same size (F_4,2153_ = 4.53; p = 0.0012). Queens in untreated colonies were also more active, as determined by the proportion of time spent immobile per observation (average ± SE 28% ± 0.04, 34% ± 0.03, and 13% ± 0.02), compared to queens in 20, 50 and 100 ppb treated colonies (63% ± 0.1, 58% ± 0.06, and 37% ± 0.05) in 1500-, 3000-, and 7000-bee colonies, respectively. Queen immobility showed greater dose-dependent responses as colony size decreased: significant differences in queen immobility were observed among treatments in 1500- and 3000-bee colonies (F_4,1213_ = 67.92; p < 0.0001) but not among treated 7000-bee colonies (F_4,1213_ = 67.92; p > 0.73) (Figure S1).

### Worker foraging behavior

Foraging activity was not recorded for colonies that contained 1500 bees. There were relatively equal proportions of foragers entering and exiting colonies within all treatment doses and colony sizes, but the total number of foragers in the 7000-bee colonies was twice the number of foragers in 3000-bee colonies. There were significantly more workers from control colonies observed entering (F_4,837_ = 50.00; p < 0.0001) and exiting the colony (F_4,837_ = 44.46; p < 0.0001) compared to colonies that received imidacloprid. Differences among treatments in foraging activity among 3000-bee colonies were dose-dependent while in the 7000-bee colonies there were no differences in foraging rate among the treatments (Figure S2).

### Worker hygienic behavior

**Figure 2:**
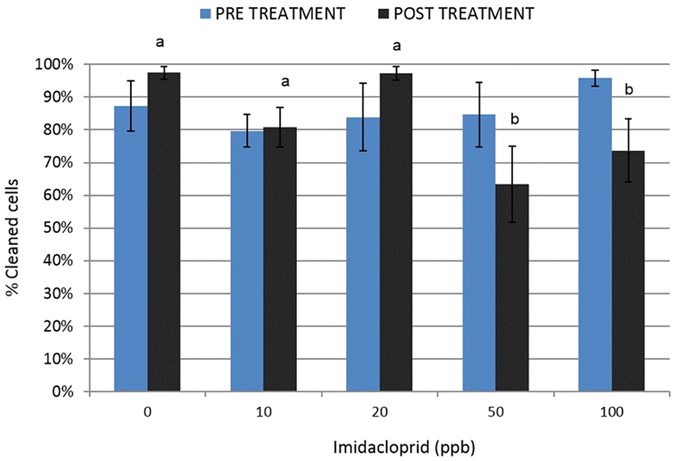
Hygienic behavior assays or percent (SE) of freeze-killed brood removed in 24 hours by worker honey bees from “7000-bee” colonies before and after chronic imidacloprid exposure (dose: F_4, 18_ = 4.5; p = 0.01). Different letters denotes significant statistical differences at α < 0.05 among treatment levels for post treatments only (no significant differences found in pre-treatment assay (F_4,18_ = 0.3; p = 0.9). Results indicate significantly lower hygienic behavior in higher treatments (50 & 100 ppb) only. Hygienic behavior was used as a measure to assess worker activity inside the hive, and is defined as the ability of worker bees to detect and remove diseased and mite-infested brood thereby limiting within-colony transmission of pathogens and parasites.

In-hive activity was assessed by performing a freeze-killed brood test, an assay for hygienic behavior, which is a behavioral mechanism of disease and parasite resistance^38^,^39^, pre- and post- imidacloprid treatment in the larger (7000-bee) colonies only. Before imidacloprid treatment, the rate of hygienic removal of freeze-killed brood was not significantly different among all colonies and averaged between 79.7% and 95.8% (F_4,18_ = 0.32; p = 0.86). After three weeks of imidacloprid exposure, the colonies treated with 50 and 100 ppb imidacloprid displayed significantly reduced hygienic removal with 63.3% ± 11.6 and 73.7% ± 9.7 of the freeze-killed brood removed respectively, compared to 97.4% ± 1.9 (control), 80.8% ± 6.0 (10 ppb), and 97.2% ± 2.1 (20 ppb) (F_4,18_ = 4.50; p = 0.011)(Fig. 2).

### Measurements after chronic exposure (day 23)

#### Adult bee population and brood production

While adult bee populations changed over the course of the 3-week experiment, the final population sizes were not significantly different among treatment levels within each colony size (F_4,55_ = 1.42; p = 0.241) (Figure S3). At the end of the treatment period, the average (SE) number of eggs in small untreated (1500-bee) colonies was significantly higher (901 ± 126) compared to eggs in treated colonies (ranging from 222 ± 218 to 489 ± 117) of the same size (F_4,26_ = 3.02; p = 0.0398). Imidacloprid-treated colonies that contained 1500 bees showed an inconsistent number of larvae and a decrease in the number of pupae as dose increased, but differences were not statistically significant (F_4,26 _= 2.18; p = 0.104 and F_4,26_ = 0.54; p ≥ 0.710, respectively). In 3000-bee colonies, the number of eggs present was not different between control and 10 ppb treated colonies of same size, but was significantly higher in control colonies than all other treated colonies (F_4,27_ = 4.05; p = 0.0125). The number of larvae in 3000-bee colonies was only significantly different between control and 100 ppb treatments (F_4,27_ = 1.26; p = 0.31), and the number of pupae in control colonies was significantly higher than all treated hives; the only differences in number of pupae among the treated hives were observed between 20 and 100 ppb treatments (F_4,27_ = 11.74; p < 0.0001). As colony size increased, no significant differences in the number of eggs, larvae, and pupae were observed among 7000-bee treated and control colonies (F_4,23_ = 1.57; p = 0.16, F_4,23_ = 0.71; p = 0.59, F_4,23_ = 1.62; p = 0.21, respectively) (Table 1). However, the ratio of adult worker bees to pupae was dose-dependent and increased from 1.6 ± 0.5 in 10 ppb treated colonies to 4.6 ± 2.2 in 100 ppb treated colonies (untreated = 1.3 ± 0.2).

### Food stores

There were no statistical differences among any of the colonies in the number of cells containing stored nectar and honey (F_4,115_ = 1.70; p = 0.16). However untreated colonies significantly more cells containing stored pollen (F_4,115_ = 22.65; p < 0.0001) than treated colonies, except in 1500-bee colonies treated at 10 ppb. The average (SE) number of cells containing pollen in treated colonies were 61–71% (1500-bee colonies), 94–138% (3000-bee colonies), and 125–161% (7000-bee colonies) lower than pollen stores found in untreated colonies at the same population size (Table 1). In addition, significant differences among treatment levels became more prominent with colony size.

### Unused cells

All colonies were started on plastic foundation imprinted with cell bases on which bees built wax cells for brood and food storage. Unused comb, defined as unused foundation (cell bases) or empty drawn wax cells, were quantified to assess space as a limiting factor for colony development and productivity. There was significantly less unused comb in untreated 3000-bee colonies than in treated colonies of the same size, but there were fewer differences among the treated and control 1500-bee colonies and no differences in unused comb among the treated and control 7000-bee colonies (F_4,115_ = 11.72; p < 0.0001) (Table 1).

### Brood pattern

**Figure 3:**
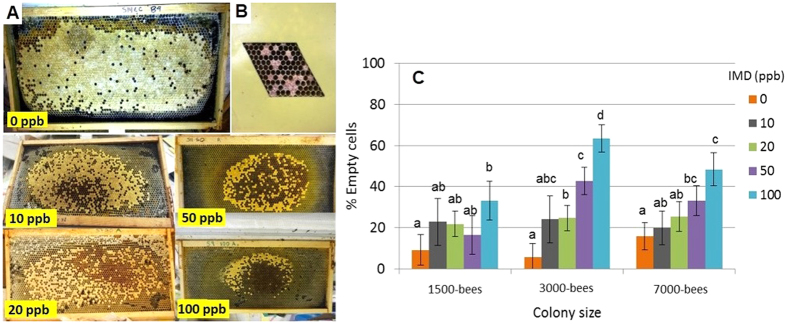
Examples of brood patterns from colonies chronically exposed to imidacloprid (0, 10, 20, 50 and 100 ppb) during brood rearing illustrating a dose-dependent effect where the amount of empty cells in a given brood area increases with treatment concentration (**A**); parallelogram containing 100 cells used to standardize brood pattern measures (**B**); and the average percentage (SE) of cells not containing pupae (empty) in a brood area of 100 cells separated by colony size (1500, 3000, and 7000 bees) and imidacloprid (IMD) dose (0, 10, 20, 50 and 100 ppb) (dose: F_4, 39_ = 10.9; p < 0.0001; colony size: F_2, 39_ = 2.1; p = 0.14; interaction effect: F_8, 39_ = 1.3; p = 0.3). Greater % of empty cells indicates worse brood patterns and overall brood health (**C**). Letters denote statistically significant differences among treatment levels within each colony size at α < 0.05. Results indicate significantly worse brood pattern (more empty cells), particularly at higher treatments (50 and 100 ppb), compared to untreated colonies.

Brood pattern (number of empty cells within a standard patch of sealed pupal cells) is an indicator of queen status and or the quality of brood care and health. The proportion of empty brood cells was significantly different among treatments (F_4,39_ = 10.93; p < 0.0001). However, there was no effect of colony size or interaction effects between dose and colony size (F_2,39_ = 2.1; p = 0.14, F_8,39_ = 1.3; p = 0.29, respectively). The overall average (SE) percentage of empty cells in control colonies was 10.2% ± 3.99, which was not significantly different from 10 ppb treated colonies (22.2% ± 6.26) but was statistically lower than 20, 50 and 100 ppb treated colonies (23.9% ± 3.86, 30.8% ± 4.6, 48.3% ± 4.7, respectively). Among the treated hives, 10, 20 and 50 ppb were not different from each other but were lower than 100 ppb treated hives (Fig. 3).

### Chemical residue analysis

Imidacloprid treatment syrup fed to colonies over three weeks was tested to confirm dosage. No imidacloprid residues were found in untreated syrup. The average residue level in syrup containing 10, 20, 50 or 100 ppb imidacloprid was 6.4 ± 4.4, 32.9 ± 2.1, 57.7 ± 6.1 and 94.2 ± 5.2 ppb, respectively (Table S2). Adult worker bees, and stored nectar collected from outside forage sources also were analyzed to estimate colony exposure. No residues were detected in bees from untreated colonies of all colony sizes and in 7000-bee colonies treated at 10 ppb. In contrast, imidacloprid was detected in bees from all other treated colonies with a positive correlation between the amount of residues detected and treatment dose within each colony size. However, residue in bees treated with 100 ppb was significantly higher than in bees from all other treatments (F_4,36_ = 9.06; p < 0.0001) (Figure S4). Imidacloprid residues detected in stored nectar generally increased with treatment dose within each colony size, however this was not significant (colony size: F_2,34_ = 1.52; p = 0.23). Low imidacloprid residues were detected in stored nectar in one 1500-bee (2.2 ng/g) and one 3000-bee (1.8 ng/g) control colony, possibly due to bees from these colonies robbing nectar from treated colonies. Stored nectar from untreated colonies exhibited significantly lower imidacloprid residues than from 50 and 100 ppb treated colonies and stored nectar from10 and 20 ppb had significantly lower residues compared to 100 ppb but not 50 ppb treated colonies (dose: F_4, 34_ = 12.68; p < 0.0001) (Figure S5).

## Discussion

This study found adverse effects of imidacloprid on honey bee queen behavior, worker bee activity, brood production, and pollen stores. Not all responses presented in a dose-dependent or monotonic manner, which is similar to other toxicological studies on neonicotinoid effects^40^,^41^. In general, effects were less evident at lower doses in larger colonies likely due to the colony’s ability to regulate resources fed to the queen and the greater number of foragers collecting outside (untreated) resources that may dilute imidacloprid levels, thus lessening potential effects. These findings elucidate the complexity of quantifying exposure effects on highly social honey bees and are in line with previous work^42^ suggesting that honey bees are less susceptible or better at detoxifying neonicotinoids compared to other bee species. Nonetheless our results indicate that small colonies may not be capable of buffering agrochemical exposure and are therefore at increased risk.

Environmentally relevant concentrations of imidacloprid, based on plant residue studies, were fed to colonies. Feeding occurred every *other* day (pulse exposure) over three weeks and the quantity of syrup fed was proportional to the colony population, but was insufficient to sustain colony development. Bees fully consumed or stored each treatment within 24 hours and all colonies were observed foraging for floral resources. Eighteen of the 216 syrup treatments, mixed over three years, were randomly tested for residues to provide an estimate of imidacloprid concentration levels fed to bees. The average residue levels in treated syrup were close to the intended dosage with the exception of 20 ppb, which was higher than intended (32.9 ± 2.1 ppb), due either to mixing error during treatment dilutions or sensitivity of analytical equipment and residue recovery rate (112.6–119.8%) by testing facilities. The imidacloprid treatments reflect residues found in the nectar and pollen of some treated agricultural crops (10–30 ppb) and ornamental plants (50, 100 ppb). Notably, most studies on seed-treated crops, such as soy, maize, and canola, reported residues at <10 ppb in nectar and pollen^43^,^44^,^45^ thus the results of this study are more relevant to colonies that are exposed to higher levels in these or other crops. Regardless, there are examples in which seed-treated maize, sunflower, and canola have yielded clothianidin residues >10 ppb in pollen and or nectar^45^,^46^. In addition to collecting potentially contaminated nectar and pollen sources, bees must collect water for thermoregulation of the colony^47^. Neonicotinoid contamination in water puddles near seed-treated maize fields during planting has been as high as 63.4 μg/L (thiamethoxam)^21^. Other studies have found guttation, or plant exudates derived from xylem collected by bees as a water source^48^, of seed-treated maize can also exhibit high levels of neonicotinoids from >10 mg/L to 346 mg/L^19^,^20^. Another consideration in estimating relevant exposure to bees is the uptake of neonicotinoids in non-target wildflowers, such as dandelions (*Taraxacum officinale*) and clovers (*Trifolium repens*, *Melilotus* spp.) that may exhibit residues from <10 ppb (dandelions near seed-treated maize) to 89–319 ppb in clover nectar when turfs were treated with clothianidin spray application^32^,^49^. These examples illustrate the immense need for more residue data to better assess environmental exposure and risk of systemic insecticides to bees.

We exposed colonies to imidacloprid and examined potential effects of *indirect* imidacloprid exposure on queen behavior and colony development. Neonicotinoids bind to nicotinic acetylcholine receptors (nAChRs) in the central nervous system activating constant transmission of nerve signals, an excitatory action that at low doses may cause hyperactivity but with increasing concentration and exposure time can cause severe tremors or paralysis in exposed bees as more nAChRs are bound^50^. Queens in treated colonies exhibited reduced fecundity likely due to imidacloprid acting directly on sensory and motor functions of the central nervous system that impacted egg-laying behavior and activity. A recent study, also suggests that neonicotinoids can compromise the viability and quantity of stored sperm in mated queens thereby further reducing queen success^31^. In nature, queen bees are indirectly exposed to environmental toxicants via trophallaxis when fed by worker nurse bees. Trophallaxis in social insects, such as ants, can attenuate toxicity of lethal toxicants particularly those that elicit delayed-action toxicity, such as neonicotinoids, by evenly distributing toxicants among nestmates and rendering them benign likely through dilution by other uncontaminated food or bodily fluids already in the gut^51^,^52^. In honey bees, the same mechanistic explanation may apply for the influence of population size on a colony’s ability to buffer pesticide exposure and toxicity. Through trophallaxis, queen bees and brood are fed royal jelly and brood food, proteinaceous glandular secretions derived from fresh and stored pollen. Stored pollen, or beebread, is eaten directly by nurse bees to stimulate production of secretions from the mandibular and hypopharyngeal glands^47^. The pathway by which contaminated food reaches queens and brood through trophallaxis might originate from the transfer of toxicants through the mandibular and hypopharyngeal glands located in the heads of nurse bees. Although little has been reported about neonicotinoid contamination in glandular secretions, imidacloprid has been detected in products containing glandular secretions such as brood food (>170 ppb acetamiprid and thiacloprid)^53^ and royal jelly (0.3–1 μg/kg) when bees were fed imidacloprid (100 ug/kg) in supplemental pollen but not syrup^35^. Imidacloprid and highly toxic metabolites (olefin and 5-hydroxy imidacloprid) have been detected in the heads of worker bees where the glands are located after they were fed ^14^C-labeled imidacloprid in syrup^54^.

In this study, indirect imidacloprid exposure through trophallaxis likely resulted in a diluted or “filtered” exposure to queens and brood, but we were unable to quantify the actual exposure levels. Individual queen bees were tested for residues but no imidacloprid or metabolites were detected possibly because individual queens (weighing < 1 g) provided insufficient sample weight to obtain results. Another possibility is that only metabolites were present in queen bees. Chemical analyses of metabolites had limits of detection of 10 and 25 ppb for olefin and 5-OH imidacloprid, respectively. Worker nurse bees attending to queens, or retinue bees, were observed feeding and grooming queens in all colonies throughout the experiment, indicating it is unlikely that reduced egg-laying was the result of poor queen attendance but was rather due to some physiological effect from exposure to imidacloprid and/or metabolites.

Chemical residue analysis of adult worker bees, and stored nectar or honey collected from inside comb cells after the chronic exposure period provided confirmation of imidacloprid exposure and contamination of food stores within the colony. For each colony size, imidacloprid detection in worker bees increased with treatment dose as expected. However, residue levels were lower than the intended dose, particularly in 7000-bee colonies, possibly due to greater numbers of foragers able to collect outside (untreated) resources and social nestmate interactions (trophallaxis) mediating or diluting exposure levels.

The combination of observed responses and chemical analysis indicates that colony size was a significant factor in reducing the toxicity and degree of affliction in treated colonies but only between the smaller (1500- and 3000-bees) and larger (7000-bees) colonies. Queens from 7000-bee colonies exhibited more gradual and graded (dose-dependent) responses, laid twice as many eggs and travelled greater distances per observation compared to the smaller colonies of the same treatment further supporting the hypothesis that imidacloprid concentrations were diluted within the larger colonies (Fig. 1). Few differences were observed in egg-laying rates and inactivity of queens between 1500- and 3000-bee colonies indicating that the smaller two sizes were not very different from each other. In epidemiological terms, social network interactions that comprise organizational immunity^55^,^56^ against pathogen transmission may be extended to pesticide exposure and attenuation of toxicity. Organizational immunity has the effect of isolating infected or intoxicated individuals through reduced social interactions and spatial segregation of diseased bees^55^,^56^. Though little is understood about the triggers and mechanisms of organizational immunity, the induction of detoxification through increased activity of enzymes in individual honey bees is negatively correlated with population size^57^. Thus small colonies may rely more on social isolation and metabolic detoxification of older foraging bees to avoid transmission of toxicants to younger hive bees that are more sensitive to pesticides and have lower detoxification capacities^58^. In contrast, larger colonies have more workers that may bring back uncontaminated forage to directly dilute collected toxicants via trophallaxis with nestmates. It may thus be more advantageous for larger colonies to increase social interactions to attenuate toxicants rather than rely on metabolic detoxification that can be energetically costly, further reinforcing the “buffering” capacity of population size to environmental toxicants, although this hypothesis would require validation^59^.

The adverse effects on queen behavior extended to colony level effects. There was significantly less brood (eggs, larvae and pupae) and more highly disrupted brood patterns observed in colonies after chronic exposure at all doses and population sizes compared to untreated colonies. Brood production is highly correlated with the population of brood-rearing nurse bees and pollen foragers and thus is a good measure for grading colony health^60^,^61^. Brood pattern is also used to assess the health of the developing brood and the queen. “Spotty” or irregular brood patterns often indicate the presence of brood diseases, a failing queen, poor brood care and or limited pollen^60^,^62^. Brood care (nursing frequency and duration) of young larvae (<4 days) is strongly correlated with the amount of pollen in the hive^62^. During times of pollen deficits, older larvae (>4 days) receive preferential feedings while younger larvae are more likely to be cannibalized to compensate for the protein shortage^63^. In this study, imidacloprid exposure had the strongest effect on the amount of pollen stored in the hive, particularly in the larger (3000- and 7000-bee) colonies likely because the amount of brood and the demand for pollen was greater. Untreated colonies had on average (SE) 4.3% ± 0.34 of all cells containing stored pollen, 6–17 times the amount compared to all treated hives, which had <2% of all cells containing pollen (10 ppb: 0.9% ± 0.51; 20 ppb: 1.5% ± 0.32; 50 ppb: 0.6% ± 0.35; and 100 ppb: 0.5% ± 0.36). Preferential cannibalism of young larvae due to pollen deficits may explain the high variation observed in larvae compared to eggs and pupae among treatments. Another explanation for lower amounts of brood in treated colonies is the potential direct toxicity due to imidacloprid exposure via contaminated brood food, which can alter the physiology and development of larvae^64^. The overall effects on brood production and pattern in this study were likely caused by a combination of factors, including effects on queen behavior, direct toxicity from contaminated food, reduced brood care and lack of pollen, but it is unclear which factors had the greatest impact on brood development.

A number of studies have demonstrated adverse effects of neonicotinoid exposure on foraging behavior in bees^12^,^30^,^65^,^66^. In our study, significantly lower foraging activity was observed in 3000- and 7000-bee colonies exposed to neonicotinoids, regardless of dose. Given there were no statistical differences in the initial and final worker bee populations it is likely that low pollen stores in treated colonies (61–161% less than in untreated colonies) was due to exposed bees being too intoxicated to forage efficiently or not stimulated to forage at all. Even with similar population sizes, the treated colonies were set back severely in brood production and pollen stores compared to untreated colonies. Although the colonies in our study were smaller than typical field colonies, colonies containing 4500 and 9000 worker bees can produce more brood per adult bee than colonies containing 17,000 and 35,000 bees^62^. Therefore population size was not a limiting factor for brood production and rearing capacity. Our findings suggest that treated colonies may appear healthy (based on population size) but may actually be performing poorly in normal colony functions based on brood and pollen stores, which have long-term consequences for colony survival and may be better indicators of colony productivity (pollination services) and health^61^. In addition, in-hive activity (hygienic behavior) in 7000-bee colonies was disrupted at 50 and 100 ppb treatments. Worker bees with impaired hygienic behavior may have been unable to detect dead brood or were motor-impaired and possibly inactive, similar to foragers and queens in treated colonies. Impaired hygienic performance could affect the colonies ability to prevent within-colony and apiary transmission of pests and pathogens, potentially making colonies exposed to neonicotinoids at high levels more susceptible to robbing by other bees, disease, and parasites^66^,^67^,^68^.

Interpretation of the environmental relevance of our findings and colony fate may require additional studies on full-sized field colonies (≥30,000 workers) and longer observation periods to determine whether queens and colonies can recover from short-term exposures. However, this study highlights the importance of mitigating neonicotinoid exposure when honey bee colonies are at low population sizes such as in early spring when colonies are small due to normal winter losses or when surviving colonies are divided by splitting the population among daughter colonies to prevent swarming. In addition, commercially available “packages” containing small populations from 7000–10000 worker bees are purchased early in spring to replace dead colonies. Small colonies, as shown in our data, which are unable to buffer or dilute neonicotinoid exposure are most vulnerable to queen effects. Risk-mitigation options should focus on reducing exposure risks when colonies are at their lowest population size due to season or management practices, for example in the early spring when risk of exposure to seed-treatment dust is at its highest during planting^44^. This study provides a mechanistic explanation for how sub-lethal effects of neonicotinoids may impair short-term colony functioning, and offers insights into potential effects of imidacloprid exposure on long-term colony survival^10^. The results have implications for promoting bee health because they offer a potential explanation for queen failures, which have been identified as a precursor to colony mortality in commercial beekeeping operations^9^.

## Methods

### Experimental colonies and treatments

Worker bees and sister queens were removed from healthy field colonies located in Chaska, MN to set up the experimental observation hives located at the University of Minnesota campus in Saint Paul, MN (approximately 64 kms away). Field colonies, requeened with queen cells each time, were used to stock observation hives multiple times each summer between May and August yielding three or four replicate studies each year over three years (2012–2014).

**Figure 4:**
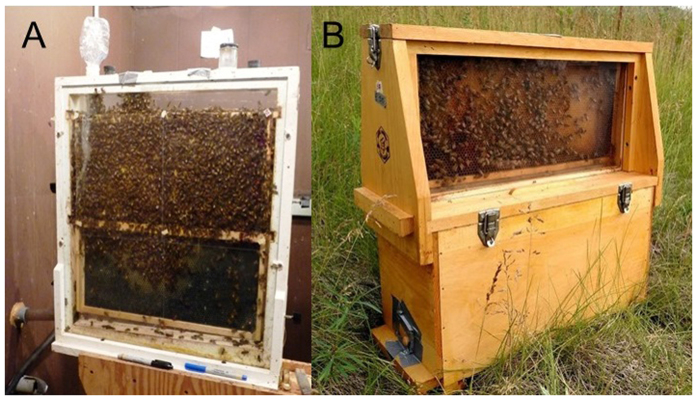
Observation hives used for 1-frame and 2-frame experimental hives containing 1500 and 3000 worker bees and a laying queen, respectively (**A**). Ulster (U501) observation hive for 5-frame experimental hives containing roughly 7000 worker bees and a laying queen. The frame containing the laying queen was placed in the upper level during observation periods while the other four frames and a feeder remained in the lower portion of the box. The entrance of the hive is located in the lower level and is shown taped closed in this picture (**B**).

One, 2, and 5 frame observation hives were established with wooden Langstroth-type deep frames and undrawn plastic foundation, and for each replicate were given a laying queen and roughly 1500, 3000, or 7000 workers estimated by weight of worker bees (0.5, 1, and 2 lbs., respectively, or 0.23, 0.45, and 0.91 kg). Colonies were provided with 2 to 4 grams of pollen supplement and sugar syrup (1:1) for 1-2 days before treatment. Smaller colonies (1500- and 3000-bees) were placed in glass-walled observation hives (Fig. 4A). The larger colonies (7000-bees) were placed in Ulster observation hives (Brushy Mountain Bee Supply, NC) containing a bottom box holding 4 standard frames and a division board feeder where treatment syrup was provided (Fig. 4B). The early morning of observation days, the fifth frame, containing the queen, was placed in an upper section made of clear Plexi-glass and separated from frames below by a queen excluder. All observation hives were housed in sheds maintained at constant temperature and relative humidity (23–25 °C and 70%). Additionally, all hives contained an entrance leading to the outside allowing bees to freely forage around some agricultural fields and urban residential neighborhoods.

After the pretreatment period and when egg laying was confirmed, each colony was randomly assigned an imidacloprid treatment (0, 10, 20, 50, and 100 ppb) provided in 50% sucrose syrup. Colonies were given proportional amounts of sucrose solution containing imidacloprid: 80, 160 and 320 mL for 1500-, 3000-, and 7000-bee colonies, respectively. Syrup was replenished every other day for 3 weeks. Syrup quantities were designed to supplement, but not sustain, the colonies so bees were required to freely forage on other resources. Stock solutions of imidacloprid (100 ppm) were prepared using 99.5 ± 0.5% technical grade imidacloprid purchased from Chem Service, Inc (PS-2086) dissolved with agitation in 50% sucrose overnight. Stock solutions were prepared every two weeks and treatment solutions were prepared every week. Samples of treatment solutions (3–6 per dose) were randomly selected and tested for residue concentrations to confirm accuracy of dosage.

The number of experimental colonies ranged from 8–20 per treatment and totaled 79 colonies over three years. Queen absconding events, where the entire colony left the hive, occurred in four smaller colonies (1500- and 3000-bees) treated at 10 ppb and one 7000-bee colony treated at 100 ppb. All colonies that absconded or had queen events were removed from the experiment, accounting for smaller sample sizes in some treatment groups.

### Measurements during chronic exposure (day 1–22)

Queen behaviors were measured through two 15-minute observations made every day for 1500- and 3000-bee colonies, and every other day for 7000-bee colonies to minimize disturbance. Queen observations from the morning (7–11 am) and afternoon (12–4 pm) bouts were averaged to account for any changes in hive activity due to weather and outside temperature. Queen activity was monitored by tracing the queen’s travel path with a felt-tipped pen onto acetate sheets placed over each observation hive, and egg-laying rate was quantified by recording the position and number of eggs laid. The distance travelled (cm) by the queen was then quantified using a digital plan measure tool (Scale Master Pro model 6025). Immobility in queens, or the time spent “resting” was also measured and defined as when the queens were not moving or grooming themselves and did not include when queens were being groomed or fed by nurse bees.

In addition to queen activity, the behaviors of adult worker bees were observed. Foraging activity was measured by recording the number of workers entering and exiting the entrance of each 3000- and 7000-bee colony during one-minute observations twice a day. Observations made on foraging behavior in 1500-bee colonies were limited and therefore removed from the experiment. Hygienic behavior was used as a measure to assess worker activity inside the hive, and is defined as the ability of worker bees to detect and remove diseased and mite-infested brood thereby limiting within-colony transmission of pathogens and parasites^38^,^39^. Hygienic behavior was measured only in 7000-bee colonies pre- and post-imidacloprid treatment. Hygienic behavior was measured using a freeze-killed brood assay, in which a 3-inch (7.6 cm) polyvinyl chloride tube was gently pushed into a section of comb containing a large area of sealed pupal cells (taken from non-experimental field colonies). The number of empty cells was counted and recorded before pouring 400 ml of liquid nitrogen to freeze-kill roughly 160 pupae. The frame was then temporarily put into 7000-bee colonies and the proportion of pupae completely or partially removed from the cells was quantified after 24 hours to assess hygienic behavior. The removal of freeze-killed brood is correlated with the removal of diseased and parasitized brood^38^,^39^.

### Measurements *after* chronic exposure (day 23)

After three weeks of imidacloprid exposure each colony was anaesthetized using carbon dioxide and placed in a −20 °C freezer. Brood production was assessed by counting all frame cells containing eggs, larvae or pupae. Brood pattern was assessed by placing a parallelogram (containing 100 cells) over 3-4 areas of sealed pupae within the colony and quantifying the average proportion of empty cells not containing pupae for each colony (Fig. 3B). Food stores, or the number of cells completely or partially filled with nectar or honey and pollen, was also quantified. Final adult worker population was determined by using the average weight of 10-subsets of ten individual bees to estimate the total number of adult workers from the total weight of the worker bee population.

### Chemical residue analyses

Treatment syrups (3 ml) were collected after solutions were made up and immediately stored in a −80 °C freezer. A total of eighteen samples of imidacloprid treatment syrup (3–6 per treatment level and colony size) were collected at random across all years. Adult worker bees (3 g) and stored comb nectar (3 ml) from three colonies per treatment and size were collected after the experiment was completed. Samples were analyzed for residues of imidacloprid and metabolites olefin and 5-hydroxy (OH) imidacloprid by the US Environmental Protection Agency Analytical Chemistry Branch (ACB) in Washington DC and the USDA Agricultural Marketing Service, National Science Laboratory (AMS-NSL) in Gastonia NC using the QuEChERS (Quick, Easy, Cheap, Effective, Rugged, and Safe) (AOAC OMA 2007.01) pesticide extraction method.

The analytical method performed by EPA-ACB used acetonitrile (15 ml) and water (10 ml) in the presence of magnesium sulfate MgSO_4_ (6 g) to extract samples. Aliquots of the extracts were passed through a C_18_SPE (1 g) and the eluents were concentrated to near dryness. For fatty sample matrices, (whole bees and stored pollen), partitioning against hexane (7 ml) was employed to remove lipids prior to the C_18_ SPE cleanup. The sample extracts were redissolved in deuterated imidacloprid internal standard solution and passed through a syringe filter. The samples were then analyzed with liquid chromatography coupled with tandem mass spectrometry detection (LC-MS/MS). The USDA-AMS-NSL laboratory extracted samples using an acetonitrile and water solution to test against certified standard reference materials and analyzed with LC-MS/MS utilizing the parent and confirmatory ions of (imidacloprid, olefin, and 5-OH imidacloprid) analytes of interest with limits of detection of 1, 10 and 25 ppb, respectively.

### Statistics

Data collected during and after the chronic exposure period were normally distributed based on quantile-quantile plots and analyzed using a mixed-effects ANOVA where imidacloprid dose, time, and colony size were treated as fixed effects and replicate and year were random effects using [SAS] software, [version University Edition] and R statistical software [version 3.2.0]^69^. Fixed effects were analyzed using a mixed effects model rather than as continuous variables in a regression model for three reasons. First, fitting as factors avoided imposition of specific curve forms across all treatments. Second, an ANOVA permits comparison among specific treatments of interest in pairwise comparisons. Finally, we focus on concentrations administered as treatments without calculating internal concentrations for each bee that would be more amenable to a regression approach. When examining the effect of time (week) on egg-laying, distance travelled and time spent immobile by queen bees, a first-order autocorrelation structure was used within the ANOVA to account for temporal dependence. Where significant treatment effects existed, mean separation procedures were performed using Tukey HSD at a significance of α = 0.05.

## Additional Information

**How to cite this article**: Wu-Smart, J. and Spivak, M. Sub-lethal effects of dietary neonicotinoid insecticide exposure on honey bee queen fecundity and colony development. *Sci. Rep.* **6**, 32108; doi: 10.1038/srep32108 (2016).

## Supplementary Material

Supplementary Information

## Figures and Tables

**Table 1:** Least square means (±SE) of brood production at each developmental stage, food stores (nectar and pollen) in comb cells, and number of unused cells in “n” number of 1500-, 3000-, and 7000-bee colonies exposed to imidacloprid (0, 10, 20, 50, 100 ppb) for three weeks.

Colony size (# bees)	ppb	n	Eggs	Larvae	Pupae	Nectar	Pollen	Unused
1500	0	6	901 ± 126 a	884 ± 174 a	1262 ± 280 a	325 ± 177 a	235 ± 39 a	927 ± 934 a
	10	2	222 ± 218 b	16 ± 301 b	1219 ± 486 a	150 ± 306 a	93 ± 66 ab	4860 ± 1474 b
	20	7	489 ± 117 b	385 ± 161 b	1017 ± 260 a	305 ± 163 a	125 ± 35 b	2849 ± 788 ab
	50	6	432 ± 126 b	519 ± 174 a	988 ± 280 a	534 ± 177 a	40 ± 38 b	1912 ± 933 ab
	100	6	423 ± 126 b	351 ± 174 b	710 ± 280 a	283 ± 177 a	51 ± 38 b	3218 ± 933 ab
3000	0	6	1085 ± 140 a	520 ± 131 a	3428 ± 314 a	848 ± 279 a	471 ± 45 a	4396 ± 1027 a
	10	2	561 ± 242 ab	453 ± 226 a	1474 ± 544 bc	408 ± 484 a	109 ± 78 bc	10113 ± 1778 b
	20	8	512 ± 121 b	355 ± 113 a	1836 ± 272 b	773 ± 242 a	169 ± 39 b	7934 ± 889 b
	50	6	397 ± 140 b	368 ± 131 a	1146 ± 314 bc	509 ± 279 a	68 ± 45 bc	9264 ± 1027 b
	100	6	428 ± 140 b	119 ± 131 b	558 ± 314 c	511 ± 279 a	28 ± 45 c	10426 ± 1027 b
7000	0	6	2360 ± 340 a	2244±654 a	6830 ± 1057 a	4845 ± 900 a	1859 ± 264 a	14994 ± 2649 a
	10	4	964 ± 416 b	1239 ± 801 a	4038 ± 1294 ab	4027 ± 1102 a	203 ± 324 b	22121 ± 3245 a
	20	5	1486 ± 372 ab	2531 ± 716 a	4791 ± 1158 ab	4067±986 a	426 ± 289 b	19488 ± 2902 a
	50	5	1746 ± 372 ab	2069 ± 716 a	3295 ± 1158 b	3407 ± 986 a	217 ± 289 b	22603 ± 2902 a
	100	4	1489 ± 416 ab	1076 ± 801 a	3645 ± 1294 ab	4123 ± 1102 a	217 ± 324 b	22784 ± 3245 a

Letters denote statistically significant differences among treatment levels within each colony size at α < 0.05.

## References

[b1] Morse R. A. & Calderone N. W. The value of honey bees as pollinators of U.S. crops in 2000. Bee Culture Magazine. 128, 1–16 (2000).

[b2] Thapa R. B. Honeybees and other insect pollinators of cultivated plants: a review. J. Inst. Agric. Anim. Sci. 27, 1–23 (2006).

[b3] vanEngelsdorp D. . Colony collapse disorder: a descriptive study. PLosONE 4, e6481 (2009). 10.1371/journal.pone.0006481 PMC2715894 19649264

[b4] Lee K. V. . A national survey of managed honey bee 2013–2014 annual colony losses in the USA. Apidologie 46, 1–14 (2015).

[b5] Laurent M., Hendrikx P., Ribiere-Chabert M. & Chauzat M.-P. A pan-European epidemiological study of honeybee colony losses 2012–2014. EPILOBEE 2, 1–44 (2016).

[b6] Kozak P. . Canadian Association of Professional Apiculturists (CAPA) National Survey Committee (2014) Statement on Honey Bee Wintering Losses in Canada. 1–5 (2014).

[b7] Kevan P. G. & Phillips T. P. The economic impacts of pollinator declines: an approach to assessing the consequences. Conserv. Ecol. 5, 8–25 (2001).

[b8] Sánchez-Bayo F. . Are bee diseases linked to pesticides?-A brief review. Environ. Int. 89–90, 7–11 (2016). 10.1016/j.envint.2016.01.009 26826357

[b9] vanEngelsdorp D., Tarpy D. R., Lengerich E. J. & Pettis J. S. Idiopathic brood disease syndrome and queen events as precursors of colony mortality in migratory beekeeping operations in the eastern United States. Prev. Vet. Med. 108, 225–233 (2013). 22939774 10.1016/j.prevetmed.2012.08.004

[b10] Bryden J., Gill R. J., Mitton R. A. A., Raine N. E. & Jansen V. A. A. Chronic sublethal stress causes bee colony failure. Ecology Letters 16, 1463–1469 (2013). 24112478 10.1111/ele.12188 PMC4299506

[b11] Cresswell J. E. A meta-analysis of experiments testing the effects of a neonicotinoid insecticide (imidacloprid) on honey bees. Ecotoxicology 20, 149–157 (2010). 21080222 10.1007/s10646-010-0566-0

[b12] Gill R. J., Ramos‐Rodriguez O. & Raine N. E. Combined pesticide exposure severely affects individual- and colony-level traits in bees. Nature 491, 105–108 (2012). 23086150 10.1038/nature11585 PMC3495159

[b13] Goulson D. REVIEW: An overview of the environmental risks posed by neonicotinoid insecticides. J. Appl. Ecol. 50, 977–987 (2013).

[b14] Carreck N. L. & Ratnieks F. L. W. The dose makes the poison: have “field realistic” rates of exposure of bees to neonicotinoid insecticides been overestimated in laboratory studies? J. Apicult. Res. 53, 607–614 (2014).

[b15] Chagnon M. . Risks of large-scale use of systemic insecticides to ecosystem functioning and services. Environ. Sci. Pollut. Res. 22, 119–134 (2015). 10.1007/s11356-014-3277-x PMC4284381 25035052

[b16] Goulson D. Neonicotinoids impact bumblebee colony fitness in the field; a reanalysis of the UK’s food & environment research agency 2012 experiment. PeerJ 3, e854 (2015). 25825679 10.7717/peerj.854 PMC4375969

[b17] Mason R., Tennekes H., Sanchez-Bayo F. & Jepsen P. U. Immune suppression by neonicotinoid insecticides at the root of global wildlife declines. J. Environ. Immunol. Toxicol. 1, 3–12 (2013).

[b18] van der Sluijs J. P. . Conclusions of the worldwide integrated assessment on the risks of neonicotinoids and fipronil to biodiversity and ecosystem functioning. Environ. Sci. Pollut. Res. 22, 148–154 (2014). 10.1007/s11356-014-3229-5 PMC4284366 25296936

[b19] Girolami V. . Translocation of neonicotinoid insecticides from coated seeds to seedling guttation drops: a novel way of intoxication for bees. J. Econ. Entomol. 102, 1808–1815 (2009). 19886445 10.1603/029.102.0511

[b20] Tapparo A. . Rapid analysis of neonicotinoid insecticides in guttation drops of corn seedlings obtained from coated seeds. J. Environ. Monit. 13, 1564–1568 (2011). 21509402 10.1039/c1em10085h

[b21] Samson-Robert O., Labrie G., Chagnon M. & Fournier V. Neonicotinoid-contaminated puddles of water represent a risk of intoxication for honey bees. PLoS ONE 9, e108443 (2014). 25438051 10.1371/journal.pone.0108443 PMC4249843

[b22] Jeshke P., Nauen R., Schindler M. & Elbert A. Overview of the Status and Global Strategy for Neonicotinoids. J. Agr. Food Chem. 59, 2897–2908 (2011). 20565065 10.1021/jf101303g

[b23] Doering J., Maus C. & Schoening R. Residues of Imidacloprid WG 5 in Blossom and Leaf Samples of Apple Trees After Soil Treatment in the Field. Application: 2003, Sampling: 2004. *Bayer CropScience AG Report. **No**. **G201819*** (2004).

[b24] Dively G. & Kamel. A. Insecticide residues in pollen and nectar of a cucurbit crop and their potential exposure to pollinators. J. Agric. Food Chem. 60, 4449–4456 (2012). 22452667 10.1021/jf205393x

[b25] Doering J., Maus C. & Schoening R. Residues of Imidacloprid WG 5 in Blossom Samples of Rhododendron sp. after Soil Treatment in the Field. Application: Autumn 2003, Sampling: 2004. *Bayer CropScience AG Report. **No**. **G201820*** (2004).

[b26] Doering J., Maus C. & Schoening R. Residues of Imidacloprid WG 5 in Blossom and Leaf Samples of Amelanchier sp. after Soil Treatment in the Field. Application: 2003, Sampling: 2004 and 2005. *Bayer CropScience AG Report. **No**. **G201799*** (2005).

[b27] Doering J., Maus C. & Schoening R. Residues of Imidacloprid WG 5 in Blossoms and Samples of Cornus mas after soil treatment in the field. Application 2003, Sampling: 2005. *Bayer CropScience AG. Report. **No**. **G201801*** (2005).

[b28] Desneux N., Decourtye A. & Delpuech J.-M. The sublethal effects of pesticides on beneficial arthropods. Annu. Rev. Entomol. 52, 81–106 (2007). 16842032 10.1146/annurev.ento.52.110405.091440

[b29] Decourtye A. . Imidacloprid impairs memory and brain metabolism in the honeybee (Apis mellifera L.). Pestic. Biochem. Phys. 78, 83–92 (2004).

[b30] Yang E. C., Chuang Y. C., Chen Y. L. & Chang L. H. Abnormal foraging behavior induced by sublethal dosage of imidacloprid in the honey bee (Hymenoptera: Apidae). J. con. Entomol. 101, 1743–1748 (2008). 10.1603/0022-0493-101.6.1743 19133451

[b31] Williams G. R. . Neonicotinoid pesticides severely affect honey bee queens. Sci. Rep. 5, 14921 (2015). 26459072 10.1038/srep14621 PMC4602226

[b32] Larson J. L., Redmond C. T. & Potter D. A. Assessing insecticide hazard to bumble bees foraging on flowering weeds in treated lawns. PLoS ONE 8, e66375 (2013). 23776667 10.1371/journal.pone.0066375 PMC3680470

[b33] Scholer J. & Krischik V. Chronic exposure of imidacloprid and clothiandin reduce queen survival, foraging, and nectar storing in colonies of Bombus impatiens. PLoS ONE 9, e91573 (2014). 24643057 10.1371/journal.pone.0091573 PMC3958374

[b34] Whitehorn P. R., O’Connor S., Wackers F. L. & Goulson D. Neonicotinoid pesticide reduces bumble bee colony growth and queen production. Science 336, 351–352 (2012). 22461500 10.1126/science.1215025

[b35] Dively G. P., Embrey M. S., Kamel A., Hawthorne D. J. & Pettis J. S. Assessment of chronic sublethal effects of imidacloprid on honey bee colony health. PLoS ONE 10, e0118748 (2015). 25786127 10.1371/journal.pone.0118748 PMC4364903

[b36] Laycock I., Lenthall K. M., Barratt A. T. & Cresswell J. E. Effects of imidacloprid, a neonicotinoid pesticide, on reproduction in worker bumble bees (Bombus terrestris). Ecotoxicology 21, 1937–1945 (2012). 22614036 10.1007/s10646-012-0927-y

[b37] Sandrock C. . Impact of chronic neonicotinoid exposure on honeybee colony performance and queen supersedure. PLoS ONE 9, e103592 (2014). 25084279 10.1371/journal.pone.0103592 PMC4118897

[b38] Rothenbuhler W. Behavior genetics of nest cleaning behavior in honeybees. I. Response of four inbred lines to disease killed brood. Anim. Behav. 12, 578–583 (1964).

[b39] Spivak M. Honey bee hygienic behavior and defense against Varroa jacobsoni. Apidologie 27, 245–260 (1996).

[b40] Caron-Beaudoin É. & Sanderson J. T. Effects of neonicotinoids on promoter-specific expression and activity of aromatase: implications for the development of hormone-dependent breast cancer. Cancer Microenviron 3, e1216 (2016).

[b41] Azevedo-Pereira H. M. V. S., Lemos M. F. L. & Soares A. M. V. M. Effects of imidacloprid exposure on *Chironomus riparius* Meigen larvae: Linking acetylcholinesterase activity to behavior. Ecotox Environ Safe 74, 1210–1215 (2011). 10.1016/j.ecoenv.2011.03.018 21511337

[b42] Rundlöf M. . Seed coating with a neonicotinoid insecticide negatively affects wild bees. Nature 521, 77–80 (2015). 25901681 10.1038/nature14420

[b43] Cutler G. C. & Scott-Dupree C. D. Exposure to clothianidin seed-treated canola has no long-term impact on honey bees. J. Econ. Entomol. 100, 765–772 (2007). 17598537 10.1603/0022-0493(2007)100[765:etcsch]2.0.co;2

[b44] Krupke C. H., Hunt G. J., Eitzer B. D. Andino G. & Given K. Multiple routes of pesticide exposure for honey bees living near agricultural fields. PLoS ONE 7, e66375 (2012). 10.1371/journal.pone.0029268 PMC3250423 22235278

[b45] Schmuck R., Schöning R. Stork A. & Schramel O. Risk posed to honeybees (Apis mellifera L, Hymenoptera) by an imidacloprid seed dressing of sunflowers. Pest. Manage. Sci. 57, 225–238 (2001). 10.1002/ps.270 11455652

[b46] Laurent F. M. & Rathahao E. Distribution of C14 imidacloprid in sunflowers (Helianthus annuus L.) following seed treatment. J. Agr. Food Chem. 51, 8005–8010 (2003). 14690387 10.1021/jf034310n

[b47] Seeley T. D. The wisdom of the hive: the social physiology of honey bee colonies. Cambridge, MA : Harvard University Press (1995).

[b48] Joachimsmeier I., Pistorius J., Heimbach U., Schenke D. & Kirchner W. Water collection by honey bees- how far will foragers fly to use water sources like guttation drops? A first distance trial using cerals and oilseed rape. 11th Symposium of the ICP-BR Bee Protection Group, Wageningen (The Netherlands) *Hazards of Pesticides to Bees*, *Nov 2011. Julius*-*Kühn*-*Archiv*, **437** (2012).

[b49] Botías C. . Neonicotinoid residues in wildflowers, a potential route of chronic exposure for bees. Environ. Sci. Technol. 49, 12731–12740 (2015). 26439915 10.1021/acs.est.5b03459

[b50] Suchail S. Guez D. & Belzunces L. P. Discrepancy between acute and chronic toxicity induced by imidacloprid and its metabolites in Apis mellifera. Environ Toxicol Chem 20, 2482–2486 (2001). 11699773 10.1897/1551-5028(2001)020<2482:dbaact>2.0.co;2

[b51] Klotz J. H. & Reid B. Oral toxicity of chlordane, hydramethylnon, and imidacloprid to free-foraging workers of Camponotus pennsylvanicus (Hymenoptera: Formicidae). J. Econ. Entomol. 86, 1730–1737 (1993).

[b52] Rondeau G. . Delayed and time-cumulative toxicity of imidacloprid in bees, ants and termites. Sci. Rep. 4, 1-8-(2014). 10.1038/srep05566 PMC4081892 24993452

[b53] Giroud B. A. V., Vulliet E., Wiest L. & Bulete A. Trace level determination of pyrethroid and neonicotinoid insecticides in beebread using acetonitrile-based extraction followed by analysis with ultra-high-performance liquid chromatography-tandem mass spectrometry. J. Chromatogr. A 1316, 53–61 (2013). 24120025 10.1016/j.chroma.2013.09.088

[b54] Suchail S., De Sousa G., Rahmani R. & Belzunces L. *In vivo* distribution and metabolisation of 14C-imdacloprid in different compartments of Apis mellifera L. Pest. Manage. Sci. 60, 1056–1062 (2004). 10.1002/ps.895 15532678

[b55] Cremer S., Armitage S. A. O. & Schmid-Hempel P. Social Immunity. Curr. Biol. 17, R693–R702 (2007). 17714663 10.1016/j.cub.2007.06.008

[b56] Stroeymeyt N., Pérez B. C. & Cremer S. Organisational immunity is social insects. Curr. Opin. Insect Sci. 3, 1–15 (2014). 32846736 10.1016/j.cois.2014.09.001

[b57] Smirle M. J. The influence of colony population and brood rearing intensity on the activity of detoxifying enzymes in worker honey bees. Physiol. Entomol. 18, 420–424 (1993).

[b58] Smirle M. J. & Winston M. L. Detoxifying enzyme activity in worker honey bees: an adaptation for foraging in contaminated ecosystems. Can. J. Zool. 66, 1938–1942 (1988).

[b59] Straub L., Williams G. R., Pettis J., Fries I. & Neumann P. Superorganism resilience: eusociality and susceptibility of ecosystem service providing insects to stressor. Curr. Opin. Insect Sci. 12, 109–112 (2015).

[b60] Harbo J. R. Effect of population size on brood production, worker survival and honey gain in colonies of honeybees. J. Apicult. Res. 25, 22–29 (1986).

[b61] Eckert C. D., Winston M. L. & Ydenberg R. C. The relationship between population size, amount of brood, and individual foraging behaviour in the honey bee Apis mellifera L. Oecologia 97, 248–255 (1994). 10.1007/BF00323157 28313936

[b62] Schmickl T. & Crailsheim K. How honeybees (Apis mellifera L.) change their broodcare behaviour in response to non-foraging conditions and poor pollen conditions. Behav. Ecol. Sociobiol. 51, 415–425 (2002).

[b63] Schmickl T. & Crailsheim K. Cannibalism and early capping: strategy of honeybee colonies in times of experimental pollen shortages. J. Comp. Physiol. A 187, 541–547.53 (2001). 11730301 10.1007/s003590100226

[b64] Derecka K. . (2013). Transient exposure to low levels of insecticide affects metabolic networks of honeybee larvae. PLoS ONE 8, e68191.60 (2001). 23844170 10.1371/journal.pone.0068191 PMC3699529

[b65] Henry M. . A common pesticide decreases foraging success and survival in honey bees. Science 336, 348–350 (2012). 22461498 10.1126/science.1215039

[b66] Alburaki M. . Neonicotinoid-Coated Zea mays seeds indirectly affect honeybee performance and pathogen susceptibility in field trials. PLoS ONE 10, e0125790 (2015). 25993642 10.1371/journal.pone.0125790 PMC4436261

[b67] Di Prisco G. . Neonicotinoid clothianidin adversely affects insect immunity and promotes replication of a viral pathogen in honey bees. Proc. Natl. Acad. Sci. Early Ed. 110, 18466–18471 (2013). 10.1073/pnas.1314923110 PMC3831983 24145453

[b68] Vidau C. . Exposure to sublethal doses of fipronil and thiacloprid highly increases mortality of honeybees previously infected by Nosema ceranae. PLoS ONE 6, e21550 (2011). 21738706 10.1371/journal.pone.0021550 PMC3125288

[b69] Chambers J. M., Freeny A. & Heiberger R. M. Analysis of variance; designed experiments. Chapter 5 of Statistical Models in S eds Chambers J. M. & Hastie T. J.Wadsworth & Brooks/Cole (1992).

