# Supplementary material for: Sub-lethal effects of dietary neonicotinoid insecticide exposure on honey bee queen fecundity and colony development
Source: Sci Rep. 2016 Aug 26;6:32108. doi: 10.1038/srep32108 (PMC4999797; doi:10.1038/srep32108)
Supplement: Supplementary Information [file srep32108-s1.pdf]

# Sub-lethal effects of dietary neonicotinoid insecticide exposure on honey bee queen fecundity and colony development

**Judy Wu-Smart[1*] and Marla Spivak[2]**
[1]University of Nebraska-Lincoln, Entomology, Lincoln, 68583, USA
[2]University of Minnesota, Entomology, Saint Paul, 55108, USA
[*]jwu-smart@unl.edu

## Supplementary Information:

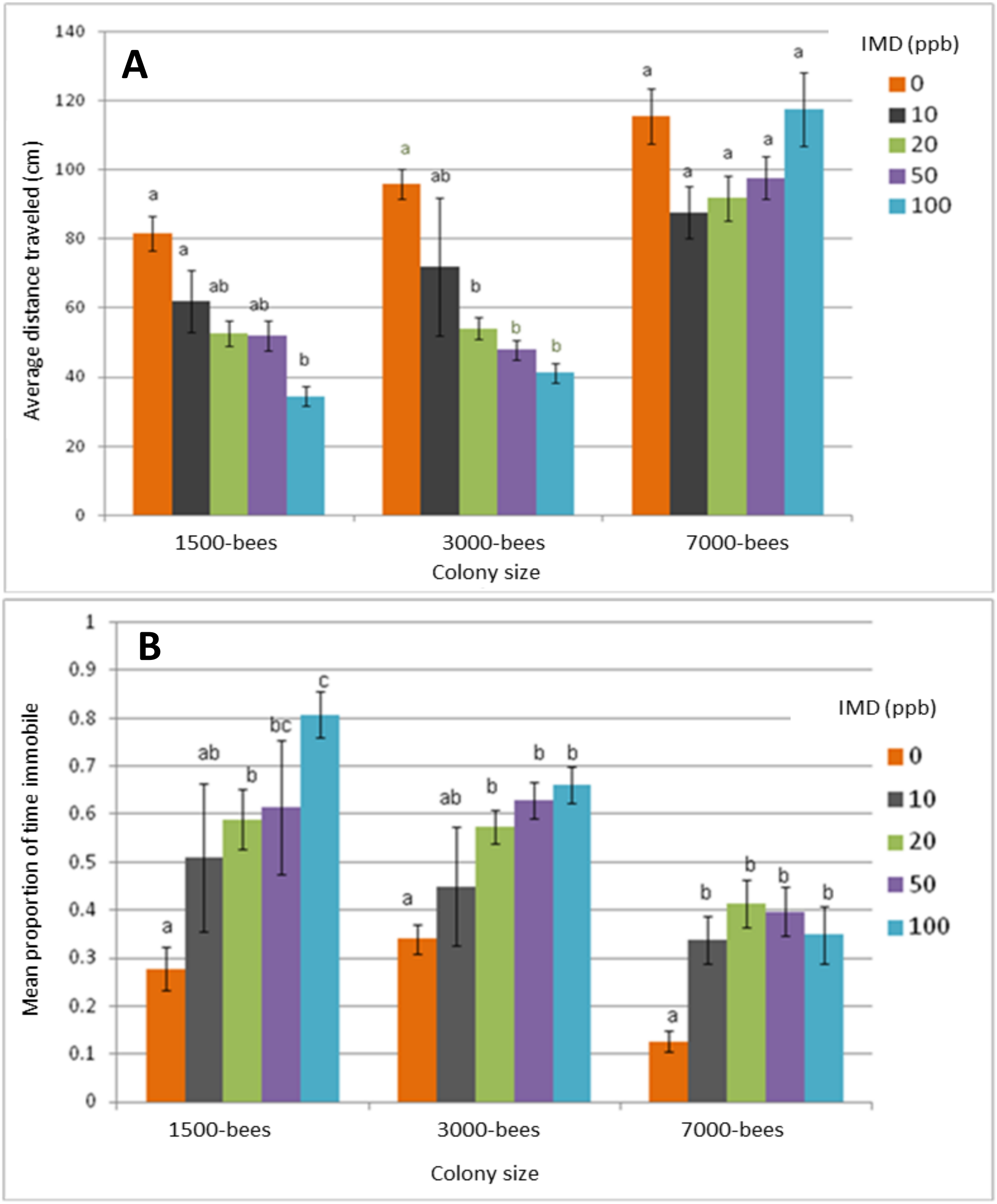


**Supplementary Figure S1.** Average (SE) distance travelled (cm) (**A**) and proportion of time immobile (**B**) by queens per 15-minute observation period in 1500-, 3000-, and 7000-bee colonies exposed to imidacloprid (IMD) treatments (0, 10, 20, 50 and 100 ppb) in 50% sucrose pooled over three weeks. Results indicate interaction effects between dose and colony size for the distance travelled by queens ((dose*size*week) interaction: $F_{16, 2153} = 1.31$; $p = 0.18$; (dose*size) interaction: $F_{8, 2153} = 4.02$; $p < 0.0001$)) and time spent immobile ((dose*size*week) interaction: $F_{16,1213} = 1.66$; $p = 0.05$; (dose*size) interaction: ($F_{8,1213} = 3.31$; $p < 0.001$)). Letters denote statistically significant differences among treatment levels within each colony size at $\alpha < 0.05$.

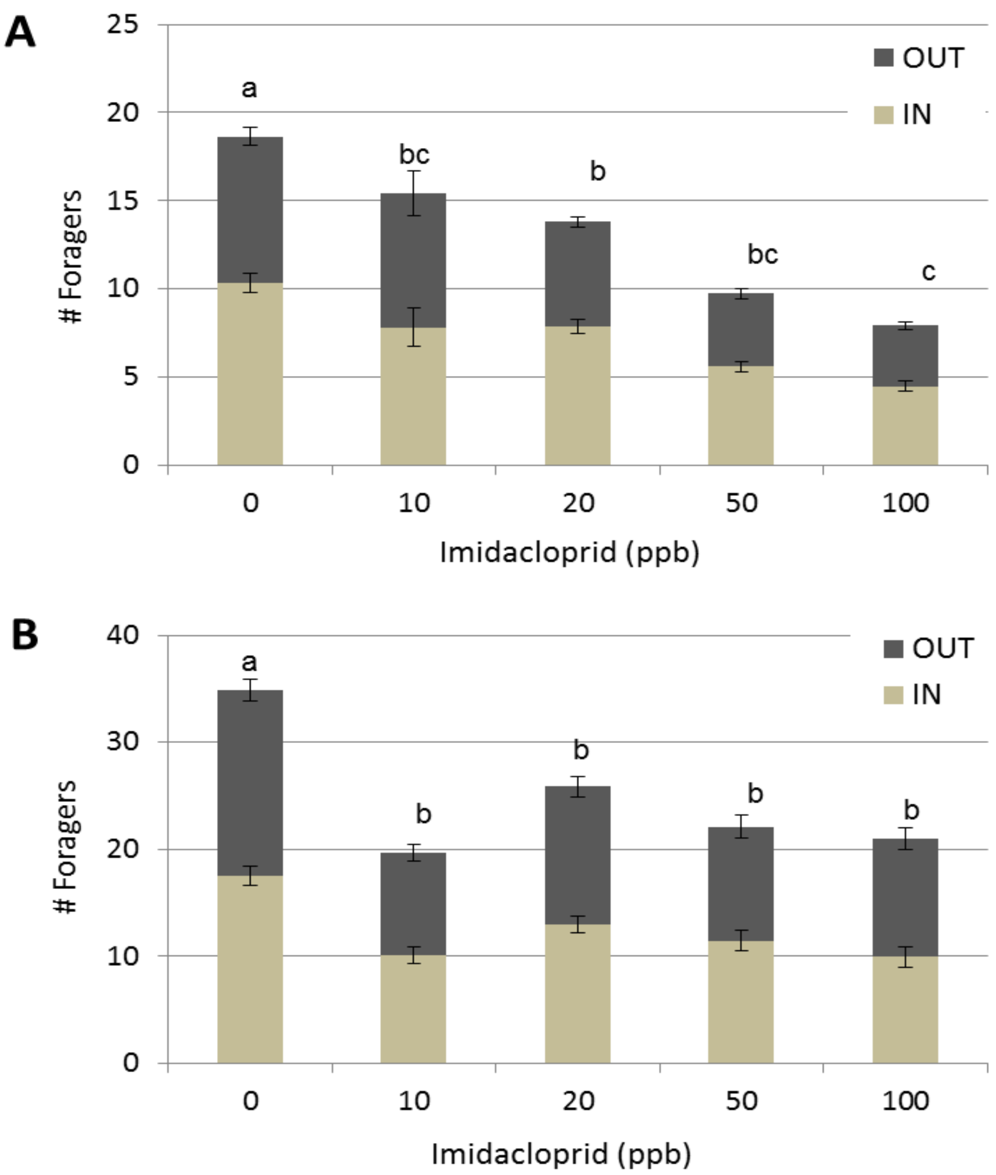


**Supplementary Figure S2** Average (SE) number of worker bees flying in (IN) and out (OUT) of (**A**) 3000-bee and (**B**) 7000-bee colonies treated with varying levels of imidacloprid (0, 10, 20, 50, and 100 ppb). Statistical analysis was separate for foragers flying IN (dose: $F_{4, 837} = 50.00$; $p < 2x10^{-16}$; colony size: $F_{1, 837} = 250.66$; $p <0.0001$; interaction effect: $F_{4, 837} = 1.19$; $p = 0.31$) and OUT (dose: $F_{4,837} = 44.46$; $p <0.0001$; colony size: $F_{1,837} = 404.85$; $p < 0.0001$; interaction effect: $F_{4, 837} = 3.03$; $p = 0.02$). Data is pooled over three weeks and letters denote statistically significant differences in the number of foragers flying both in and out among treatment levels at $\alpha < 0.05$. Results indicate a dose-dependent effect on foraging in 3000-bee colonies but not in 7000-bee colonies.

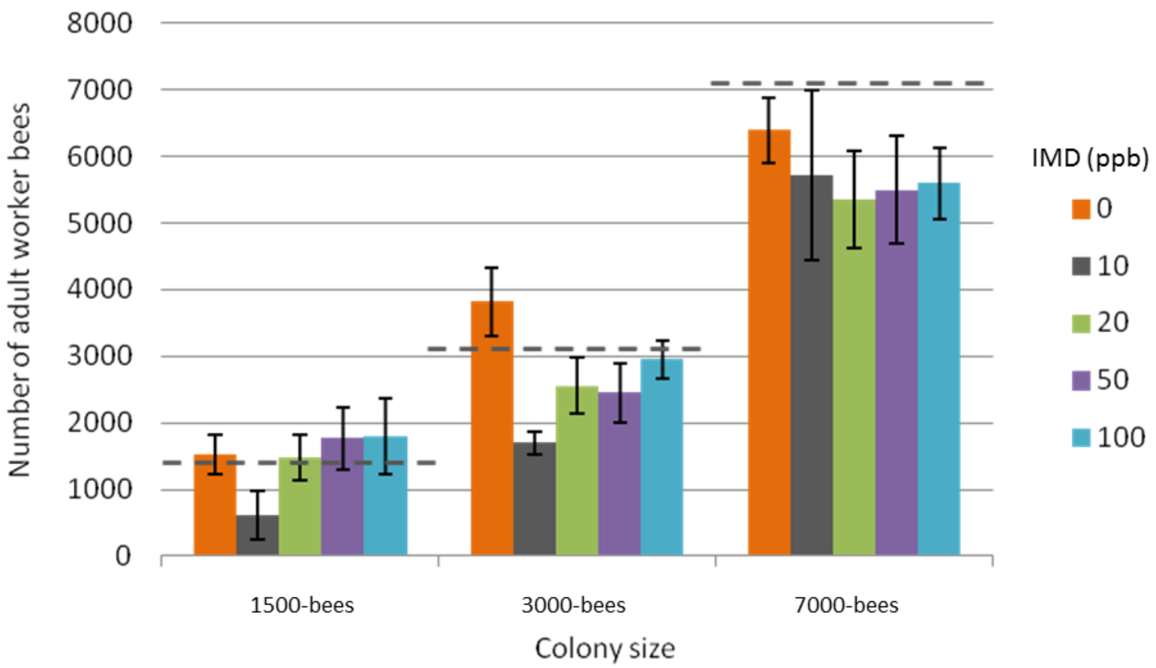


**Supplementary Figure S3.** Final adult worker bee population (±SE) after three weeks of chronic imidacloprid (IMD) exposure at varying levels (0, 10, 20, 50, and 100 ppb) in 1500-, 3000-, and 7000-bee colonies. There were no significant differences among treatment levels within all three colony sizes ($F_{4, 55} = 1.4$; $p = 0.241$). Dotted lines represent initial populations for each colony size.

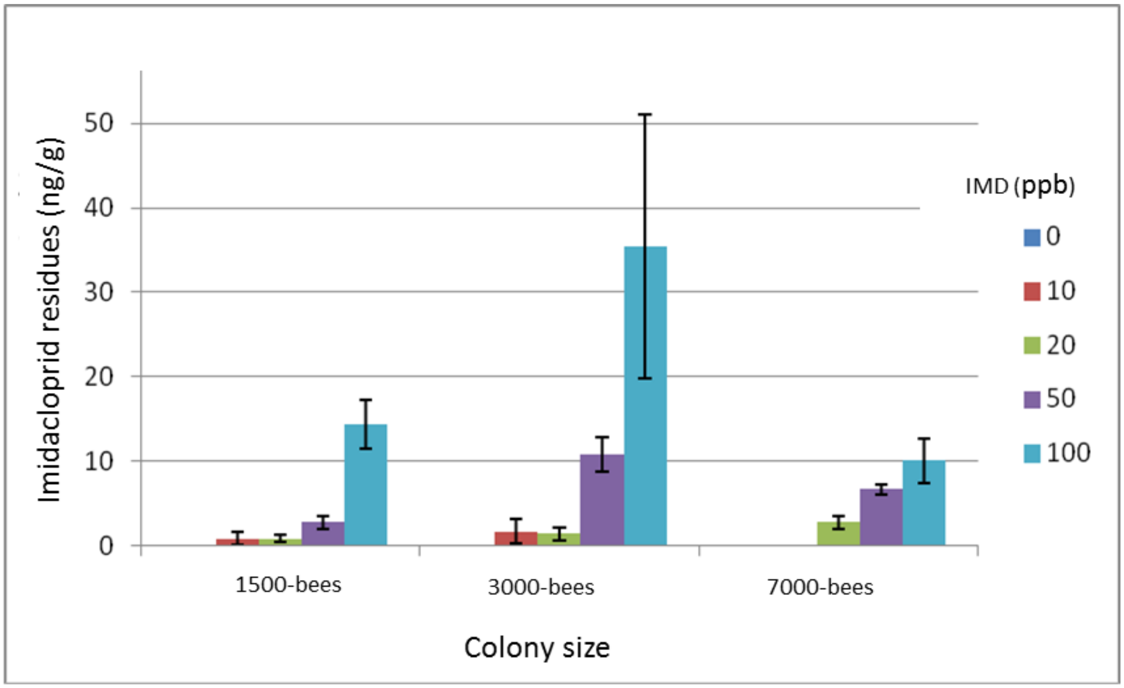


**Supplementary Figure S4.** The average (SE) level of imidacloprid residues detected in adult worker bees collected from experimental colonies of different sizes (1500-, 3000- and 7000-bees) treated at 0, 10, 20, 50, and 100 ppb imidacloprid (IMD) after three weeks of chronic exposure (dose: $F_{4, 36} = 9.06$; $p < 0.0001$; colony size: $F_{2, 36} = 2.79$; $p = 0.07$; interaction effect: $F_{8, 28} = 1.81$; $p = 0.1$). Results indicate significantly higher residues found in 100 ppb treated worker bees compared to all other treatment levels and there were no differences among 0, 10, 20, and 50 ppb treated bees.

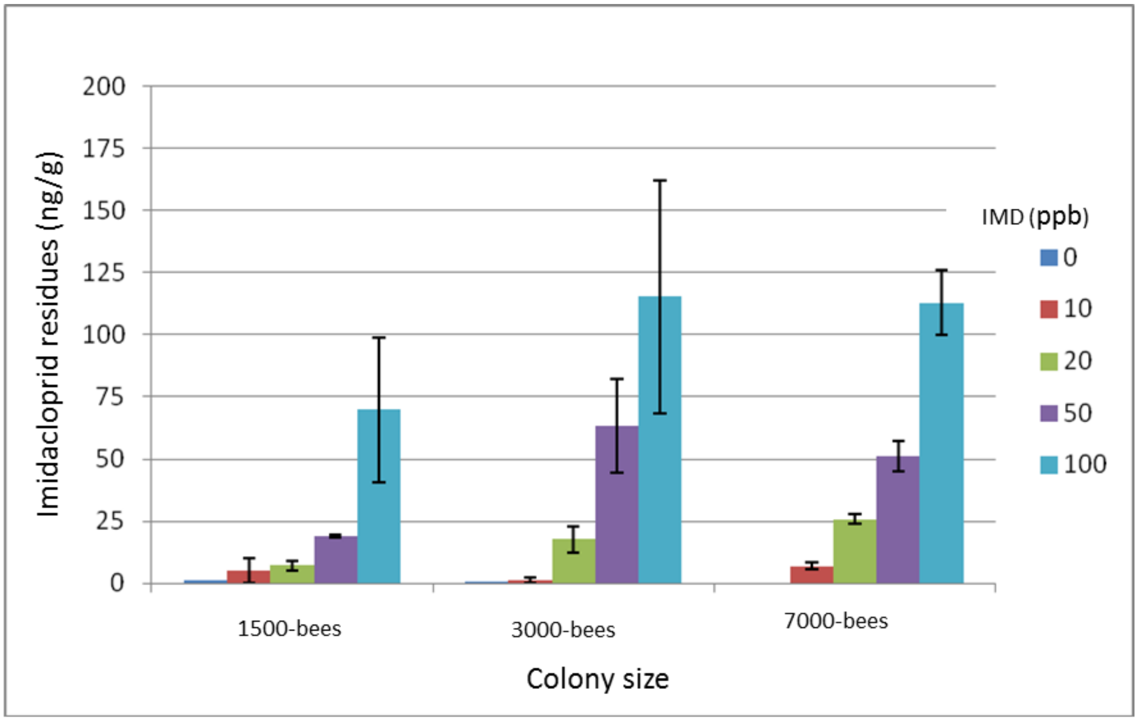


**Supplementary Figure S5.** The average (SE) level of imidacloprid residues detected in stored nectar or honey collected from comb cells inside experimental colonies of different sizes (1500-, 3000- and 7000-bees) treated at 0, 10, 20, 50, and 100 ppb imidacloprid (IMD) after three weeks of chronic exposure (dose: $F_{4, 34} = 12.68$; $p < 0.0001$; colony size: $F_{2, 34} = 1.52$; $p = 0.23$; interaction effect: $F_{8, 26} = 0.52$; $p = 0.8$). Results indicate comb nectar from untreated colonies exhibited significantly lower imidacloprid residues than 50 and 100 ppb treated colonies and comb nectar from 10 and 20 ppb had significantly lower imidacloprid residues than 100 ppb treated colonies.

| | | Imidacloprid exposure time | | | | | | | |
|---|---|---|---|---|---|---|---|---|---|
| Colony size | Dose (ppb) | pre-trt | | Week 1 | | Week 2 | | Week 3 | |
| 1500 bees | 0 | 5.17 ± 0.89 | a | 6.75 ± 0.77 | a | 6.44 ± 0.80 | a | 6.18 ± 0.76 | a |
| | 10 | 7.67 ± 1.27 | a | 5.41 ± 0.86 | ab | 2.67 ± 0.78 | b | 2.40 ± 0.91 | b |
| | 20 | 4.66 ± 0.98 | a | 3.90 ± 0.72 | b | 2.45 ± 0.76 | b | 2.84 ± 0.71 | b |
| | 50 | 5.58 ± 0.89 | a | 3.75 ± 0.77 | b | 1.69 ± 0.80 | b | 2.16 ± 0.81 | b |
| | 100 | 5.92 ± 0.89 | a | 3.61 ± 0.77 | b | 2.51 ± 0.83 | b | 2.23 ± 0.77 | b |
| 3000 bees | 0 | 6.99 ± 0.73 | a | 6.75 ± 0.45 | a | 5.41 ± 0.47 | a | 5.88 ± 0.49 | a |
| | 10 | 8.33 ± 1.62 | a | 1.68 ± 1.33 | bc | 3.18 ± 1.33 | ab | 1.47 ± 1.18 | b |
| | 20 | 7.21 ± 0.73 | a | 3.92 ± 0.43 | b | 3.12 ± 0.42 | b | 2.55 ± 0.43 | b |
| | 50 | 5.78 ± 0.73 | a | 3.07 ± 0.45 | c | 2.53 ± 0.46 | b | 2.36 ± 0.49 | b |
| | 100 | 6.49 ± 0.73 | a | 2.35 ± 0.46 | c | 2.34 ± 0.46 | b | 2.29 ± 0.47 | b |
| 7000 bees | 0 | 10.75 ± 1.20 | a | 11.07 ± 0.63 | a | 11.20 ± 0.59 | a | 8.51 ± 0.59 | a |
| | 10 | 10.19 ± 1.33 | a | 10.12 ± 0.75 | a | 5.24 ± 0.67 | bc | 4.53 ± 0.67 | b |
| | 20 | 8.92 ± 1.20 | a | 5.01 ± 0.67 | bc | 5.43 ± 0.63 | bc | 2.68 ± 0.61 | c |
| | 50 | 10.19 ± 1.25 | a | 3.69 ± 0.68 | c | 3.96 ± 0.62 | c | 3.67 ± 0.61 | bc |
| | 100 | 11.69 ± 1.33 | a | 5.48 ± 0.72 | b | 3.64 ± 0.69 | c | 3.24 ± 0.65 | bc |

**Supplementary Table S1** Least square means of queen bee egg-laying rate per 15-min observation before imidacloprid treatment (pre-trt) and during chronic exposure (week 1, 2, and 3) of imidacloprid treatment at various doses (0, 10, 20, 50, and 100 ppb) in three different colony sizes (1500-, 3000-, and 7000-bees). Different letters denotes statistically significant differences among treatment levels at $\alpha<0.05$ within each time period.

| Testing facility | Sample ID | Treatment | Sample size (g) | IMD | olefin | 5-OH IMD |
|---|---|---|---|---|---|---|
| EPA | S512 | 0 | 3 | N.D. | N.D. | N.D. |
| USDA | SS1 | 0 | 3 | N.D. | N.D. | N.D. |
| USDA | SS8 | 0 | 3 | N.D. | N.D. | N.D. |
| EPA | S514 | 10 | 3 | 16.5 | N.D. | N.D. |
| USDA | SS5 | 10 | 3 | 1.3 | N.D. | N.D. |
| USDA | SS9 | 10 | 3 | 1.3 | N.D. | N.D. |
| EPA | S518 | 20 | 3 | 32.1 | N.D. | N.D. |
| EPA | S517 | 20 | 3 | 36.9 | N.D. | N.D. |
| USDA | SS2 | 20 | 3 | 29.8 | N.D. | N.D. |
| EPA | S515 | 50 | 3 | 59.1 | N.D. | N.D. |
| EPA | S520 | 50 | 3 | 64.6 | N.D. | N.D. |
| EPA | S519 | 50 | 3 | 83.6 | N.D. | N.D. |
| USDA | SS3 | 50 | 3 | 47.7 | N.D. | N.D. |
| USDA | SS6 | 50 | 3 | 47.1 | N.D. | N.D. |
| USDA | SS10 | 50 | 3 | 44.0 | N.D. | N.D. |
| EPA | S516 | 100 | 3 | 103.8 | N.D. | N.D. |
| USDA | SS4 | 100 | 3 | 85.8 | N.D. | N.D. |
| USDA | SS7 | 100 | 3 | 104 | N.D. | N.D. |
| USDA | SS11 | 100 | 3 | 83.0 | N.D. | N.D. |

**Supplementary Table S2** Pesticide residues (ppb) of imidacloprid (IMD) and metabolites (olefin and 5-OH imidacloprid) detected in varying levels (0, 10, 20, 50, 100 ppb) of imidacloprid treatment syrup fed to experimental colonies chronically for three weeks. The limits of detection were 1, 10 and 25 ppb, for IMD, olefin, and 5-OH I.MD, respectively. (N.D.= not detected)
